# Electron Microscopy Studies of Local Structural Modulations in Zeolite Crystals

**DOI:** 10.1002/anie.202007490

**Published:** 2020-08-07

**Authors:** Qing Zhang, Alvaro Mayoral, Junyan Li, Juanfang Ruan, Viveka Alfredsson, Yanhang Ma, Jihong Yu, Osamu Terasaki

**Affiliations:** ^1^ Center for High-Resolution Electron Microscopy (CħEM) School of Physical Science and Technology ShanghaiTech University 393 Middle Huaxia Road, Pudong Shanghai 201210 China; ^2^ Institute of Nanoscience and Materials of Aragon (INMA) CSIC-University of Zaragoza 12, Calle de Pedro Cerbuna 50009 Zaragoza Spain; ^3^ State Key Laboratory of Inorganic Synthesis and Preparative Chemistry College of Chemistry International Center of Future Science Jilin University 2699 Qianjin Street Changchun 130012 China; ^4^ Electron Microscope Unit Mark Wainwright Analytical Centre The University of New South Wales Sydney NSW 2052 Australia; ^5^ Physical Chemistry Lund University P.O. Box 124 SE-22100 Lund Sweden

**Keywords:** crystallography, electron microscopy, imaging, zeolites

## Abstract

Zeolites are widely used in catalysis, gas separation, ion exchange, etc. due to their superior physicochemical properties, which are closely related to specific features of their framework structures. Although more than two hundred different framework types have been recognized, it is of great interest to explore from a crystallographic perspective, the atomic positions, surface terminations, pore connectivity and structural defects that deviate from the ideal framework structures, namely local structural modulation. In this article, we review different types of local modulations in zeolite frameworks using various techniques, especially electron microscopy (EM). The most recent advances in resolving structural information at the atomic level with aberration corrected EM are also presented, commencing a new era of gaining atomic structural information, not only for all tetrahedral atoms including point vacancies in framework but also for extra‐framework cations and surface terminations.

## Graphical abstract

**Local structure modulations** deviating from ideal framework‐type structures are important fine‐structural features within zeolite frameworks. By using (S)TEM with diffraction and imaging approaches, point, column and planar modulations can be observed in both reciprocal and real space.

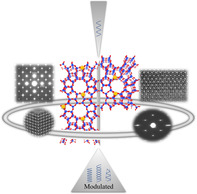

## 1 Introduction

A zeolite framework is formed by corner sharing oxygen atoms in TO_4_ tetrahedra, where the T atom is in general Si or Al. The formula of an aluminosilicate zeolite is generally described as M^*m*+^_*x*/*m*_[(AlO_2_)_*x*_(SiO_2_)_*y*_]⋅*p* H_2_O per unit‐cell or M^*m*+^_*z*/*m*_[Al_*z*_ Si_1−*z*_O_2_]⋅*p* H_2_O, where M is an exchangeable counter cation with a valence of *m*+, and *p* H_2_O is zeolitic water. The range of *x* is less than or equal to *y* and the Si/Al ratio, defined by *y*/*x* (a commonly used parameter in the zeolite community), ranges from infinity for pure silica polymorphs to 1, according to Löwenstein's rule^1^, although a few exceptions have been reported recently by B. Slater^2^. Natural zeolites, such as Sodalite (**SOD**), Faujasite (**FAU**), Mordenite (**MOR**), Cancrinite (**CAN**), Erionite (**ERI**), Heulandite/Clinoptilolite (**HEU**) and so on, have rather low framework Si/Al ratios and have been applied for sustainable agricultural usage.

Because of the diverse potential applications of zeolites in the fields of petrochemistry, catalysis, gas adsorption/separation, etc., zeolite science has been shifted from natural to synthetic materials in order to create and improve their physicochemical properties, such as internal acidity, selectivity, and thermal stability through regulation of various structural and compositional parameters.

Although there are currently 252 different framework types identified by the International Zeolite Association (IZA),^3^ only some of them are used in industry based on their outstanding properties and low cost in comparison to other alternatives. For instance, **LTA** (Si/Al=1) is used as a detergent (water softener) and desiccant due to its excellent ion exchange and water uptake properties; **FAU**, which can be crystallized with different Si/Al ratios, named zeolite X or Y, can be used for adsorption of water or organic molecules or in fluid catalytic cracking. High silica zeolites such as ZSM‐5 (**MFI**) and ZSM‐11 (**MEL**),^4^ firstly synthesized by Mobil scientists, are mainly used in the petrochemical industry owing to their high thermal stabilities and specific structural features. Both structures can be described as the simplest ordered end‐members of the pentasil zeolite family.

In order to understand the physical properties of zeolites in addition to their chemical compositions, structural information such as atomic crystallographic positions, surface terminations, pore connectivity and structural defects should be thoroughly studied. Framework‐type structures may be defined in terms of an “ideal structure” by assuming that a zeolite is a polymorph of silica, SiO_2_, with no extra‐framework cations and by taking the highest symmetry. Here, “modulation” is used to refer to structural deviations from the ideal structure in real space, in terms of type and position of T‐atoms and framework symmetry. Such features can be observed as point, columnar and planar modulations by high‐resolution scanning/transmission electron microscopy (HR‐S/TEM) images or in more specific ways, through the appearance of extra reflections, diffuse scatterings or intensity changes of reflections in electron diffraction (ED) patterns in reciprocal (momentum) space.

Electron microscopy (EM) provides a great opportunity to obtain information of structures in both real and reciprocal space from the same nano‐volume of a crystal. In the case of zeolites, the basic principle in forming a structure comes from a rigid TO_4_ tetrahedral unit, which is connected to neighboring TO_4_ units by corner‐sharing oxygen atoms. A rigid‐unit‐mode (RUM) model could be applied to describe a modulated structure (as for example structural transformation with temperature, including negative thermal expansion), where very rigid TO_4_ units would be allow to change their relative configurations of neighboring units through bending or rotation with respect their shared O atoms without breaking the T‐O‐T bonds.^5^ However, applying this model to modulated zeolite structures is still very challenging. It is too complicated to study modulated structures of zeolites by analysis of powder X‐ray diffraction (PXRD) data because of seriously inherent peak overlap and peak broadening induced by the modulation. In the case of **MFI** and **MEL** zeolites, a typical type of modulation can be described as stacking disorder of pentasil‐sheets. Perego and co‐workers introduced *i*‐type (inversion) and *σ*‐type (mirror) stacking descriptions in analysis of the PXRD results,^6^ although minor differences might still arise in fitting the fault probability parameter *p*. Approaches involving HR‐TEM and ED should be carried out with cautious treatment of multiple scattering. Nowadays, with the development and implementation of aberration correctors, S/TEM provides detailed structural information of nano‐crystals at atomic level.

**Figure 1:**
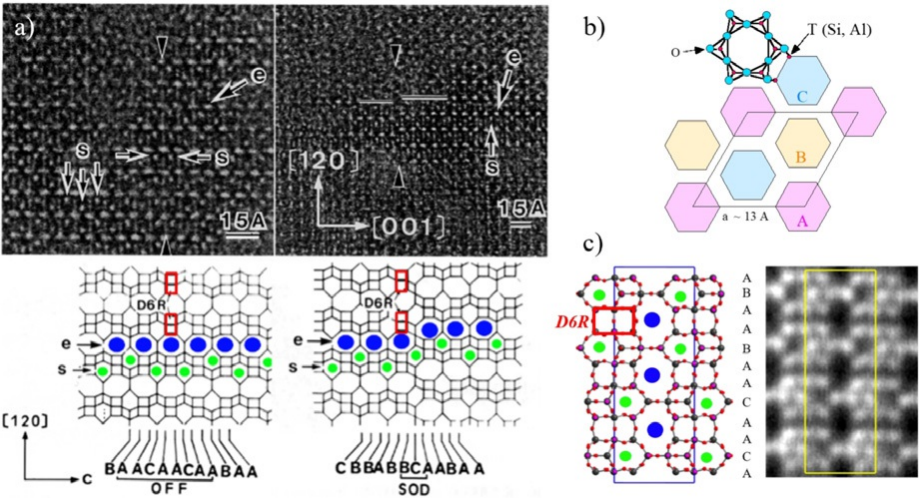
Planar modulations in ABC‐6 family. a) HRTEM images of **ERI** zeolites where **OFF** and **SOD** structure can be found with models below the images. Reproduced with permission.^7^ Copyright 1986, Elsevier. b) Projected schematic of ABC‐6. c) HR‐STEM image and the projected structural model of STA‐20. Reproduced with permission.^8^ Copyright 2017, American Chemical Society. 6Rs, 8Rs and D6Rs are marked by green dots, blue dots and red rectangles, respectively.

Although spatial resolution, due to imperfection in the EM lenses, has been a major limiting factor for a long time, it has still been possible to determine stacking sequences from HR‐TEM images taken perpendicular to the stacking direction, not only in **MFI** and **MEL** 9 but also in other families of polytypes such as the ABC‐6 family, **FAU** and **EMT** or Beta zeolites. A few illustrative examples of the power of HR‐TEM are presented in Figure 1 a. The observation of **ERI** (a zeolite belonging to the ABC‐6 family), with stacking of layers of **OFF** and **SOD**, is shown in Figure 1 a. The ABC‐6 family is one of the most important groups in zeolite science due to their small‐medium pore sizes and good thermal stabilities, which have made them in high demand for reactions such as the treatment of pollutants from combustion of vehicles (NO and CO) or reactions relevant to alternative energy sources such as methanol‐to‐olefin (MTO) conversion.^10^ The infinite set of ABC‐6 zeolites can be obtained by stacking sheets consisting of hexagons, six‐membered rings (6Rs) of TO_4_ tetrahedra, with their centres taking three different positions, A, B or C on the projection perpendicular to the sheets (Figure 1 b). In contrast to hard sphere packing, the maximum number of successive stacking of the same type is two (i.e. AA, or BB, or CC), forming double six‐membered rings (D6Rs) and eight‐membered rings (8Rs) next to each other. D6Rs, 6Rs and 8Rs are imaged as short line‐segments, small and large bright dots, respectively, in HR‐TEM images (Figure 1 a). Therefore, the corresponding frameworks (marked by red rectangles, green dots and blue dots, respectively), can be uniquely determined as shown in Figure 1 a. This is the first example in which HR‐TEM images provided direct information of the stacking sequence in a zeolite.^7^ For comparison, Figure 1 c shows STA‐20 (**SWY** framework), the most recent silicoaluminophosphate with ABC‐6 structure, which was characterized by spherical aberration (C_s_) corrected STEM together with the unit cell schematic model, representing another excellent example of stacking modulation characterization by EM.^8^

**Figure 2:**
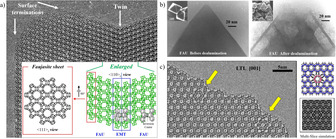
a) Intergrowth of **EMT** within two regions of **FAU** as the boundary modulation. Schematic models of twin plane and Faujasite sheet are shown as insets. Reproduced with permission.11b Copyright 1995, Elsevier. b) HRTEM and SEM images for faujasite before and after dealumination. Reproduced with permission.^13^ Copyright 1994, American Chemical Society. c) Columnar modulation in LTL which coincides with the schematic model and simulated image. Reproduced with permission.^15^ Copyright 2004, Wiley‐VCH.

Both **FAU** and **EMT** have a common structural unit, the faujasite sheet, in which all sodalite (**SOD**) cages are connected through inversion at the centre of D6R. In **FAU** and **EMT**, successive faujasite sheets are related by inversion and mirror, respectively. Figure 2 a shows the intergrowth of **EMT** within two regions of **FAU** as the planar (boundary) modulation.^11^ Viveka Alfredsson^12^ firstly observed the structure of the surface termination (incomplete **SOD** cages by removing D6R from **FAU** framework) in zeolites. Of course, the surface termination is one of the most important structure modulations from a crystal to vacuum. As for the **FAU**‐type framework, the uniqueness of its large three‐dimensional (3d) accessible volume (for molecules up to 27 % in contrast to 9.8 % and 12.7 % for **MFI** and **MEL**, respectively) makes it a special candidate for “dealumination”. As a type of point and framework modulation, dealumination has been developed to increase the strength of a framework, both in terms of internal acidity and thermal stability. As far as we know, dealumination of **FAU** (zeolite‐Y) to make it an ultra‐stable hydrophobic zeolite‐Y (USY) while keeping periodic original **FAU** framework structure was started by Sten Andersson (Lund Univ), Lars Falth (Zeol, later MuntersZeol, Lund), Tetsu Ohsuna and Osamu Terasaki (Tohoku University) together with To‐Soh (Japanese Company)^13^ to capture smelly organic compounds at high moisture preventing outlets from the factory of Tetra‐Pack (Lund) in 1980′s. Observations of structural changes in **FAU** (Figure 2 b) induced by dealumination have been reported by SEM and HR‐TEM. Furthermore, observations of zonings with different Si/Al ratios in **MOR** was reported in an oscillatory grown crystal since growth rate of a crystal depends on the ratio.^14^ Therefore, direct observation at the atomic scale of a point modulation in composition during crystal growth or dealumination (including formation of hydroxyl nest) is a really challenging research target.

In addition, columnar structural modulation in **LTL** was observed by Tetsu Oshuna^15^ (Figure 2 c). The data were recorded using a JEM‐4000EX operated at 400 kV. Figure 2 c shows the HR‐TEM image of **LTL** along [001] with columnar faults marked by yellow arrows.

In general, these few examples illustrate the richness of zeolite science from a structural perspective and how advanced EM can help to understand these materials in ways that other methodologies cannot do. In this review, we describe various structural modulations in zeolite frameworks from an EM perspective. Promising results on aberration corrected STEM are also presented as the first step to directly observe T atoms, oxygen bridges and even single extra‐framework cations, which is an essential future direction for characterization of structural modulations at the atomic level.

## 2 Structural Modulations in Zeolites

### 2.1 Basic structure of pentasil family and planar modulation in MFI/MEL

**MFI** and **MEL** zeolites are ordered end‐members within the pentasil family. They display different types of modulations, as shown below. The differences between the two structures in projection are so small that the intergrowths between them have mainly been studied by electron diffraction, based on the stacking description model between *i*‐type and *σ*‐type.^16^ Several years later, it was possible to directly observe the position and type of symmetry elements in the projected structures from HR‐TEM images of **MFI** and **MEL** through great advances in high‐quality crystal syntheses.^17^

**Figure 3:**
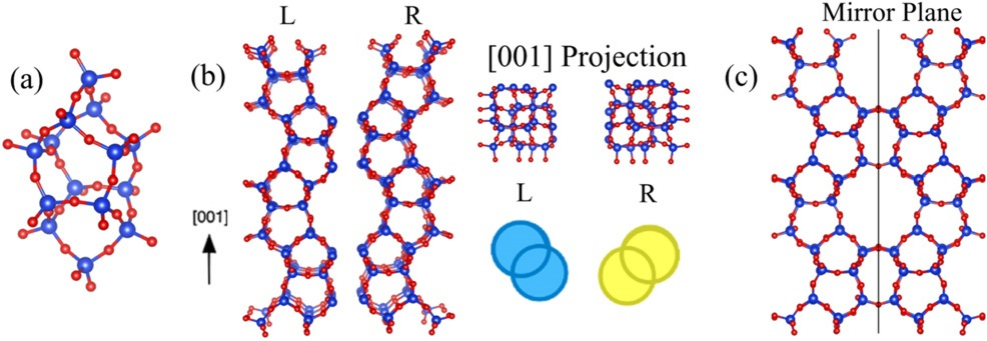
Schematic models of the pentasil structure. a) Pentasil unit. b) Pentasil chains with left‐ and right‐handed chirality. c) Pentasil sheet which is formed from the left and the right chains through mirror symmetry.

The frameworks can be described based on the pentasil‐unit, pentasil‐chain and pentasil‐sheet shown in Figure 3. The pentasil‐unit has characteristic features: eight five membered rings (5Rs) with symmetry element of 4_1_ *m*2 with the units joined through edges to form a pentasil‐chain with left‐ or right‐handed chirality along the 4_1_‐axis. These left‐ and right‐handed chains are connected through mirror symmetry to form the pentasil‐sheet, which is the basic and common structural unit of **MFI** and **MEL**.

**Figure 4:**
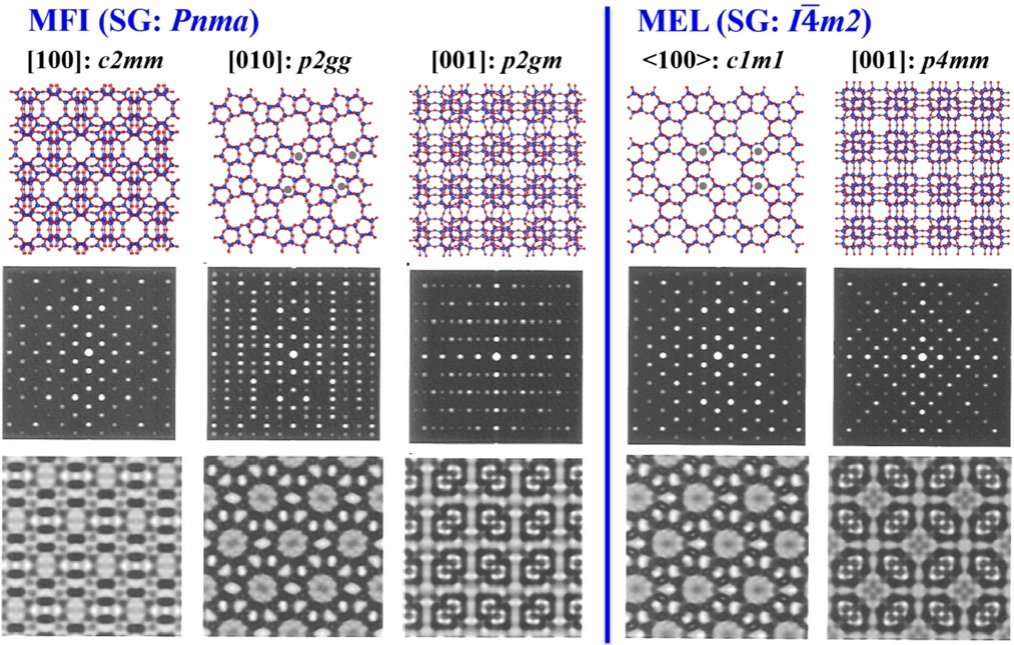
Comparison of **MFI** and **MEL** along the principal zone axes: top) frameworks, middle) simulated ED patterns, and bottom) simulated HR‐TEM images with their corresponding plane groups. Some larger five membered rings in the models are marked by dark dots to show the symmetry.

Figure 4 shows the projected framework structures of **MFI** and **MEL** with plane group symmetries along the 3 principal zones axes, [100], [010] and [001]. The main difference of **MEL** and **MFI** can be found in their projected structure **MEL** <100> and **MFI** [010], where the pentasil sheets are related by mirror or inversion centre, respectively. We note that a three‐dimensional inversion centre *i* is observed as a 2‐fold axis on the projected two‐dimensional image along [010] for **MFI**. The dots marked in the model correspond to the larger 5Rs. The corresponding simulated ED patterns and HR‐TEM images (at Scherzer focus) are shown by use of multi‐slice method for crystal thickness of 100 Å and JEM‐4000EX operated at 400 kV. For EM images with enough spatial resolution, [001] is the best direction to distinguish between **MFI** and **MEL**, as the chiral‐chains and their arrangement along the chain direction can be observed and distinguished. In an excellent work, Ohsuna and co‐workers reported the first observation of the co‐existence of both materials within the same crystal.17c

**Figure 5:**
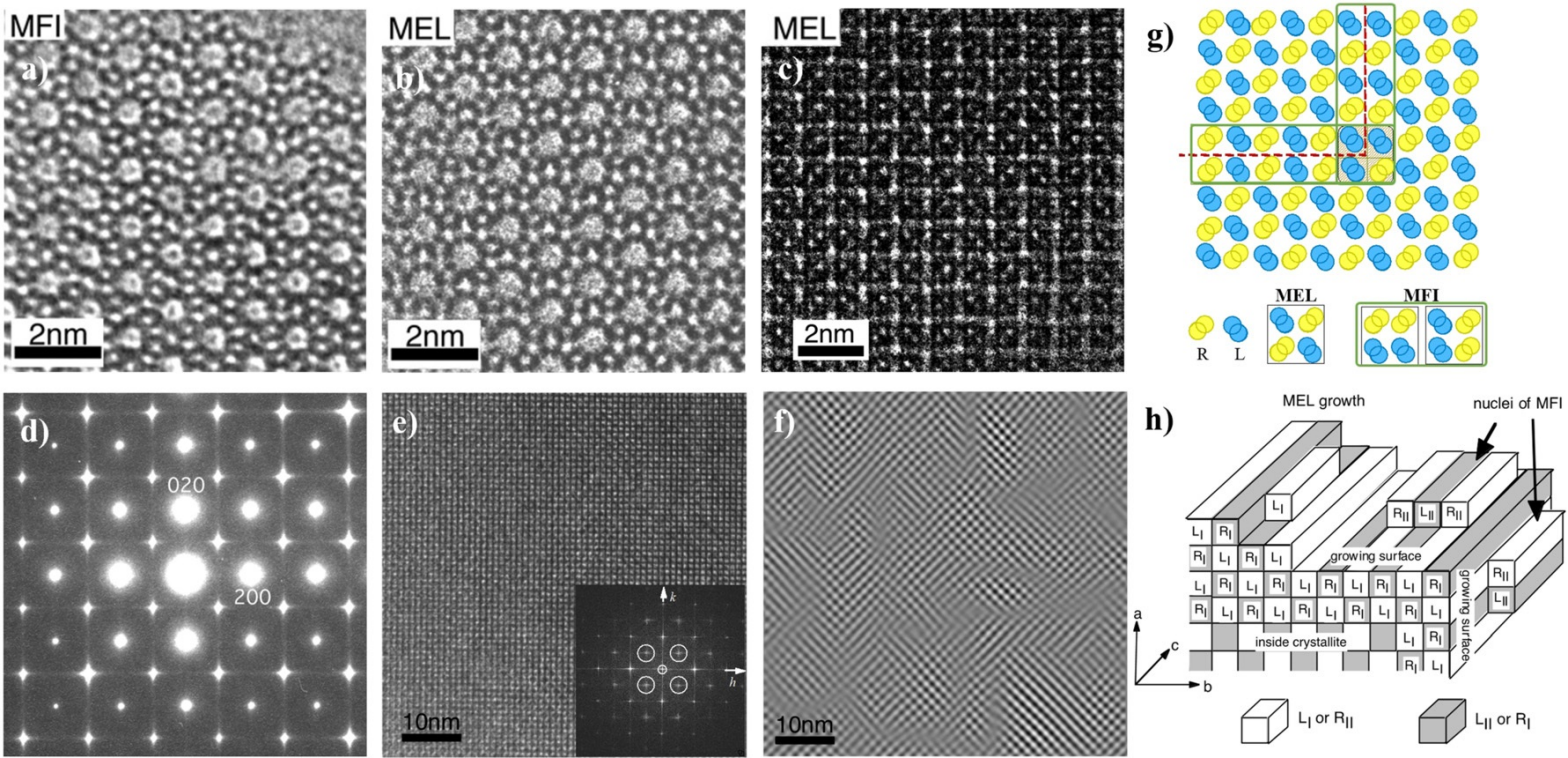
HR‐TEM images of **MFI** along [010], **MEL** along <010> and **MEL** along [001] (a–c). Planar modulations in B‐MEL looking along [001]: d) ED pattern, e) HR‐TEM image, and f) Fourier filtered HR‐TEM image, f) schematic of planar modulation using the L and R pentasil‐chains, and g) schematic model of crystal growth. in B‐MEL. Taken by JEM‐4000EX at 400 kV. Reproduced with permission.17c Copyright 1997, American Chemical Society.

Figure 5 shows the HR‐TEM images of **MFI** and **MEL** (Figure 5 a–c) and planar modulation observed in the B‐MEL system (Figure 5 d–h). Clear streaks are seen in the ED pattern of B‐MEL (Figure 5 d) running through the *hk0* reflections (*h*, *k*=2*n*+1) and the weak contrast modulation in the HR‐TEM image together with Fourier diffractogram (FD) (Figure 5 e). The Fourier filtered image using the 000 and four 110 reflections from the FD shows a hazy contrast running parallel to the (100) and (010) planes. On this basis, a model of the chain‐type related planar modulation in the framework of B‐MEL projected along the [001] direction is schematically illustrated in Figure 5 g using the L and R pentasil‐chains. Assuming that the pentasil chain is a growth unit on **MEL** crystals, a model for the growth process along (100) surfaces of the B‐MEL crystal can be proposed as shown in (Figure 5 h).

**Figure 6:**
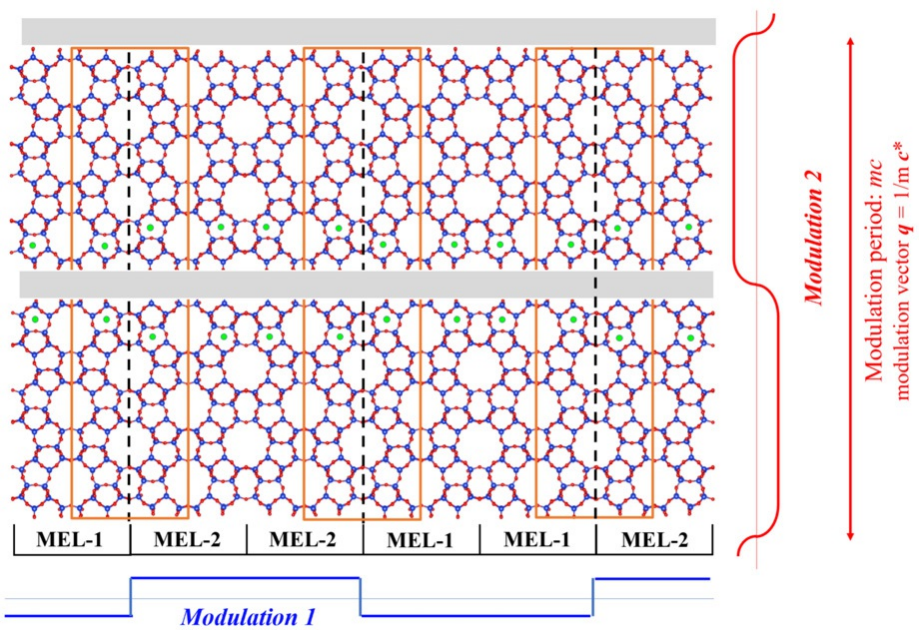
Schematic of two types of planar modulation in **MEL** frameworks. When pentasil chains are connected by changing symmetry from mirror to inversion (MEL‐1 to MEL‐2 or inverse) at a plane on dashed lines and **MFI** units in orange rectangles appear, which is Modulation 1. In this case, there is no change in atomic density as shown in sharp step function. While Modulation 2 may introduce a change in atomic density across the grey boundaries.

“Planar‐modulation models” can be further extended from [010] projection of **MEL** with a boundary parallel to (100) and (001) planes. The boundary can be either a common shared plane for (100) (Modulation 1, as in FAU/EMT, Figure 2 a) or narrow extra boundary regions (grey bands) to form smooth framework connections (001) (Modulation 2) as shown in Figure 6. The periodic existence of this planar fault can be commensurate or incommensurate with respect to the basic lattice. Incommensurate structure modulation in zeolites was first observed in SSZ‐24 (**AFI**‐type framework), in which atomic positions are incommensurately modulated along the *c*‐axis. It was analysed by RUM model.^18^ Besides, SSZ‐57 with possible incommensurately modulated structure is demonstrated as follow through Modulation 2.

**Figure 7:**
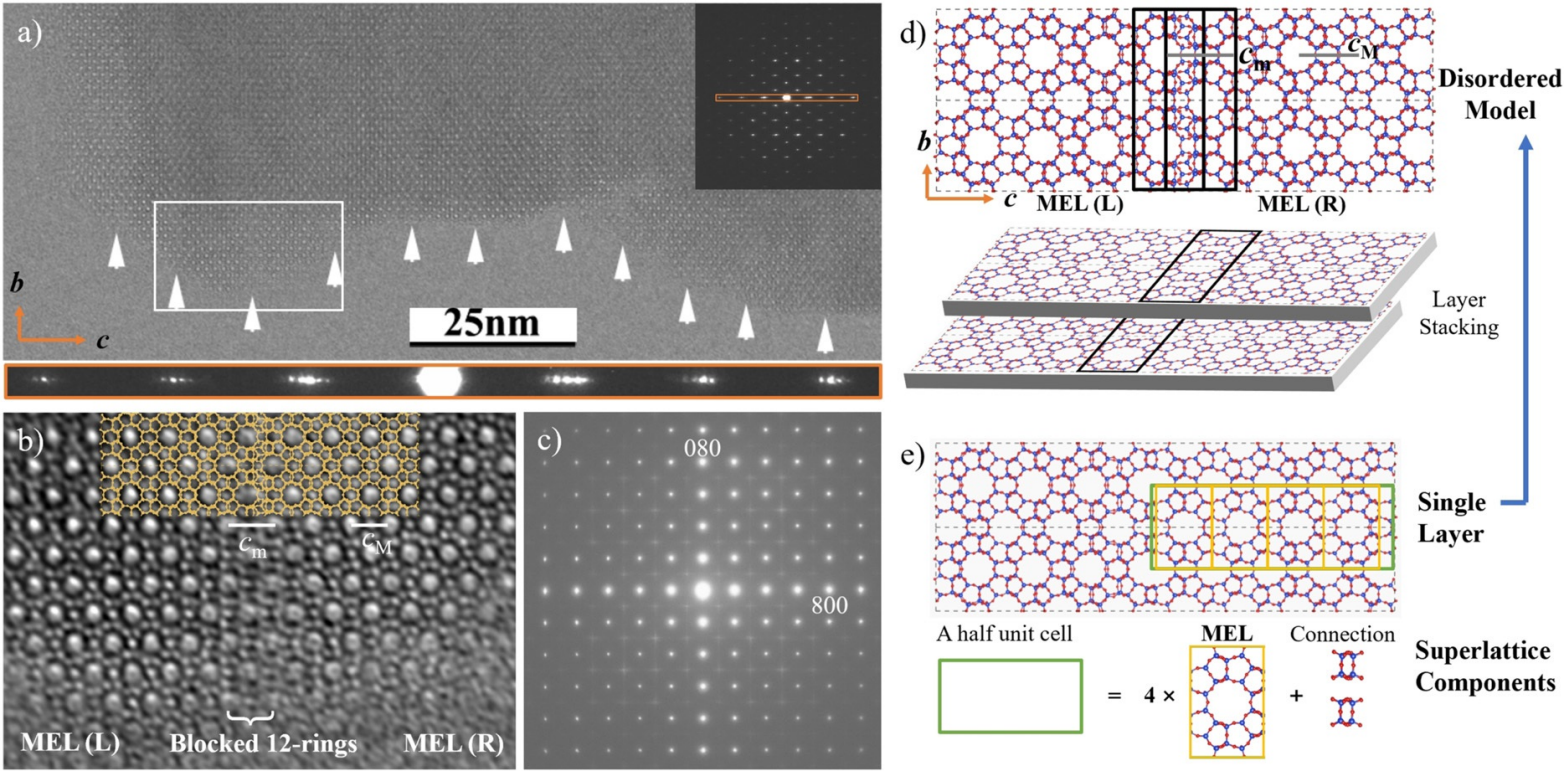
HR‐TEM images, ED pattern and structure models of SSZ‐57. a) HR‐TEM image taken along [100] and the diffraction pattern were inset with *00l* reflections. Modulation contrasts are marked by white arrows. b) Magnified image of the rectangle area in panel (a) overlaid the framework shown in panel (d). c) SAED patterns obtained from SSZ‐57 taken along [001]. d) Disordered structure model from Christian et al.^19^ and the schematic of super structure. The blocked twelve membered ring are formed by overlapping of two twelve membered rings columns marked by black rectangles. e) *b‐c* plane of the idealized structure model of single layer, which forms a super structure consisting of 8 **MEL** cells and the connection part. Reproduced with permission.^20^ Copyright 2011, Elsevier.

High silica SSZ‐57 zeolite (*SFV) was synthesized in 2003 at Chevron using the same synthesis procedure of **MEL**. SSZ‐57 shows some similarities with boron loaded MEL (B‐MEL) in terms of: (i) morphology, (ii) 4‐fold rotation symmetry along the *c*‐axis and (iii) similar diffuse streaks in the SAED pattern along [001] incidence (Figure 5 d and Figure 7 c). The structure of SSZ‐57 was solved by Baerlocher et al.^19^ in 2011 from high‐quality single‐crystal (2 μm × 2 μm × 8 μm) synchrotron X‐ray diffraction. They studied the framework structure in 4‐dimensional space (corresponding to Figure 6, Modulation 2) using Superflip algorithm. The solution was related to ZSM‐11 (**MEL**) but commensurately modulated along the *c*‐axis (*P*4*m*2, *a*=*b=*20.091 Å, *c=*110.056 Å) with modulation vector *q*=0.125 c*, yielding a structure with twelve membered ring (12R): ten membered ring (10R) ratio of 1:15. They also discussed the disordered structure as a modulated one, which is highly scholarly treated model that could explain the experimental diffraction and HR‐TEM images.^20^

The SAED pattern of SSZ‐57 taken along [100] is shown as an inset in Figure 7 a, which is very similar to that of **MEL** taken along [100] (Figure 4) except for the existence of a clear modulation along the c*‐axis (an enlarged pattern of *00l* is shown in the orange rectangle). Baerlocher et al.^19^ proposed an idealized model of SSZ‐57 (Figure 7 e) involving an eight‐fold superstructure formed by 8 **MEL** cells and two “connections” (four ring) as a unit cell. We noted, however, that periods of the contrast modulation observed in Figure 7 a show a few irregularities marked by white arrows. Due to the introduction of the “connection”, the unit cell of SSZ‐57 is larger than 8 times that of **MEL** along the *c*‐axis. The spacing of the *00l* superstructure reflections (Figure 7 a) is approximately 1/8 of the c*. Although the incommensurate modulation should be taken into consideration, this idealized model gives a good explanation of the SAED pattern of SSZ‐57.

The enlarged HR‐TEM image from the white rectangle in Figure 7 a shows two domains marked MEL(L) and MEL(R) together with (but not so well resolved due to possible overlap) a band between them that would correspond to the blocked 12Rs; for easier interpretation the model of the framework is overlaid in yellow (Figure 7 b). The period of the large bright dots in MEL (L) and MEL (R) along *c*‐axis (corresponding to the 10Rs) is given by *c_M_*, while the period in the central part is given by *c_m_*, which is slightly larger than *c_M_*.

To further interpret the HRTEM image, a structural disorder in SSZ‐57 was introduced, described by misplacement of 12R columns in the stacking of the pentasil sheets. Figure 7 d shows how the two pentasil sheets stack along the *a*‐axis, 12R columns (marked by black rectangles) blocking each other to be 10R and a distinct projected structure is produced in the overlapping region. Different intervals of large pores in the HRTEM images (*c_M_* and *c_m_* in Figure 7 b) can be explained by the insertion of the “connections” and overlapping 12Rs in this model.

Here, a simplified model with limited disorder was found to be coincident with the domain in the HRTEM image. The disorder would be more complicated in the macroscopic crystal as it may occur in two dimensions (*a*‐axis and *b*‐axis) due to the symmetry. Thus the stacking disorder along the *a*‐axis would appear also along the *b*‐axis. The diffraction pattern of SSZ‐57 along [001] (Figure 7 c) reveals homogeneous but weak diffuse streaks through *hk0* reflections (*h*, *k*=2*n*+1) and extended along the a* and b* reciprocal axes, similar to that for B‐MEL (Figure 5 d). Diffraction patterns for the SSZ‐57 structure with random disorder simulated by a Monte Carlo algorithm verify this structure model, as the results give an extremely good match to the single crystal X‐ray diffraction pattern, allowing the probability of faults to be determined in the calculated model.^19^ Thus, by using advanced crystallographic techniques and EM analysis, quasi‐ideal SSZ‐57 with an incommensurately modulated structure along *c*‐axis was successfully observed. The pore geometry is modified toward a new absorption behaviour from the structural modulation such as a three‐dimensional 10R channel system with large isolated 12R pockets, which may provide many advantages in applications of zeolites.

### 2.2 Intrinsic structural modulation in unit cell of IMF

Another good example of structural modulation in the pentasil family is the IM‐5 zeolite with IMF framework type. Because of heavily overlapped reflections and extra peaks from impurity phases in the PXRD pattern, the structure of IM‐5 remained unsolved for almost 10 years after its discovery. The structure of IM‐5 was reported in 2007 by Baerlocher et al.,^21^ using a newly developed charge‐flipping structure‐solution algorithm combining PXRD data and TEM data, and by Sun et al.21b from TEM data in 2010.

**Figure 8:**
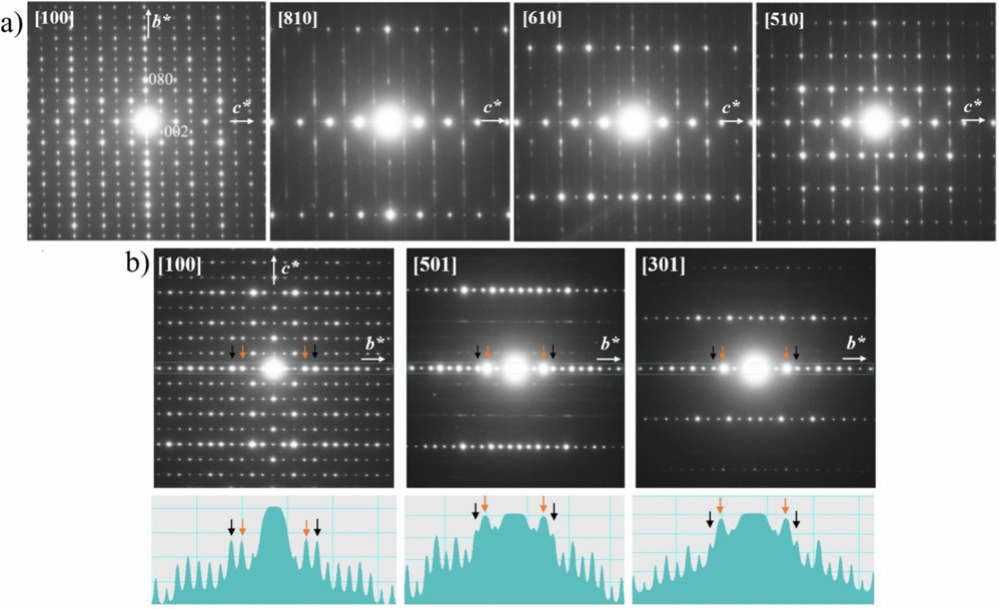
SAED patterns selected from two series of tilting calcined IMF around (a) the c*‐axis and (b) the b*‐axis. The intensity distribution of *0k0* reflections is shown at the bottom.

Three‐dimensional ED data were collected from nano single‐crystals of IM‐5 by Ruan.^22^ Two sets of a series of selected area electron diffraction (SAED) patterns were collected by tilting a crystal around the b*‐ and c*‐ axes, observing mirror symmetries perpendicular to their axes. For following discussions, some of them are shown in Figure 8 a. The reciprocal planes perpendicular to c*‐axis have diffuse intensities (Figure 8 a) while diffuse streaks are observed by a section with Ewald sphere in Figure 8 a and 8 b. However, all *0k0* reflections are sharp without streaks along the b*‐axis, which is the most heavily modulated direction. Based on these observations, the modulation can be explained as a structural unit perpendicular to the *b*‐axis. The main components of the modulation along *b* (b*) at [100] incidence, where multiple scattering is enhanced, are the 060 and 080 reflections, indicated by orange and black arrows in Figure 8 b, respectively, while the 060 reflection becomes the main component in [501] and [301] incidences. This indicates a possible three‐fold superlattice within the frameworks. Sun et al. also noticed this modulation and described it as a *pseudo* three‐fold super‐lattice.

**Figure 9:**
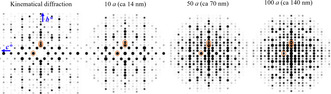
Calculated electron diffraction patterns of IM‐5 crystal with different thickness along [100] incidence under 300 kV. Phases and amplitudes of the 002, 060 and 080 reflections (orange circles in the patterns) are shown in Table 1. Dynamical diffraction patterns are simulated by eMap using the multi‐slice method based on structure data (using reflections with resolution up to 1.67 Å) taken from Baerlocher et al.21a

Possible space groups were obtained from observed 3d‐electron diffraction data: *Cmc*2_1_ (36), *C*2*cm* (40, standard setting A*ma*2), and *Cmcm* (63). Among them, only *Cmcm* has a centre of inversion. In this case, the phase of the crystal structure factor for the reflections could be either 0 or π, if the reference origin is taken at the inversion centre, while for *Cmc*2_1_ and *C*2*cm*, the phases could deviate from 0 or π. Therefore, Baerlocher et al. and Sun et al.^21^ took *Cmcm* after considering the a possibility of *C*2*cm*. However, it was found that few strong reflections in the FD of **IMF** along [100] incidence, largely deviate from 0 or π in an independent work of Ruan.^22^ In particular, the relative phases to the origin could change with increase of crystal thickness through multiple scattering. ED patterns of IM‐5 (model from IZA with *Cmcm* space group) were simulated for different thickness (Figure 9; phase information is given in Table 1), showing great differences between the kinematic condition and a rather thin sample. Additionally, phase information derived from HRTEM images is affected by experimental conditions, including deviation of electron incidence from an exact crystal zone axis. In such circumstance, it is difficult to determine the space group by phase information from the HRTEM images.

**Table 1:** Phases and amplitudes of electrons for different reflections of IM‐5, at 300 kV, calculated by eMap. Structural data is taken from Baerlocher et al.21a (Space group *Cmcm*, unit‐cell parameters *a*=1.430 nm, *b*=5.679 nm, *c*=2.029 nm).

Reflections		Kinematical^[a]^	Dynamical^[b]^ diffraction with different crystal thickness			
		diffraction	1.4 nm	14 nm	70 nm	140 nm
000	Phase (°)	0	4.5	45.2	22.5	−40.3
002	Phase (°)	0	96.2	144.0	154.0	145.7
	Amplitude	458.0	1.5	12.8	11.7	4.3
						
060	Phase (°)	180	−83.4	−28.1	−42.8	−5.4
	Amplitude	502.9	1.5	12.1	19.1	9.5
						
080	Phase (°)	0	92.5	114.0	123.2	166.3
	Amplitude	301.1	0.8	8.0	8.1	15.1

[a] The origin of lattice is set at the inversion center. [b] Phase value for dynamical scattering is not relative to the 000 beam but the incident beam for all thickness.

**Figure 10:**
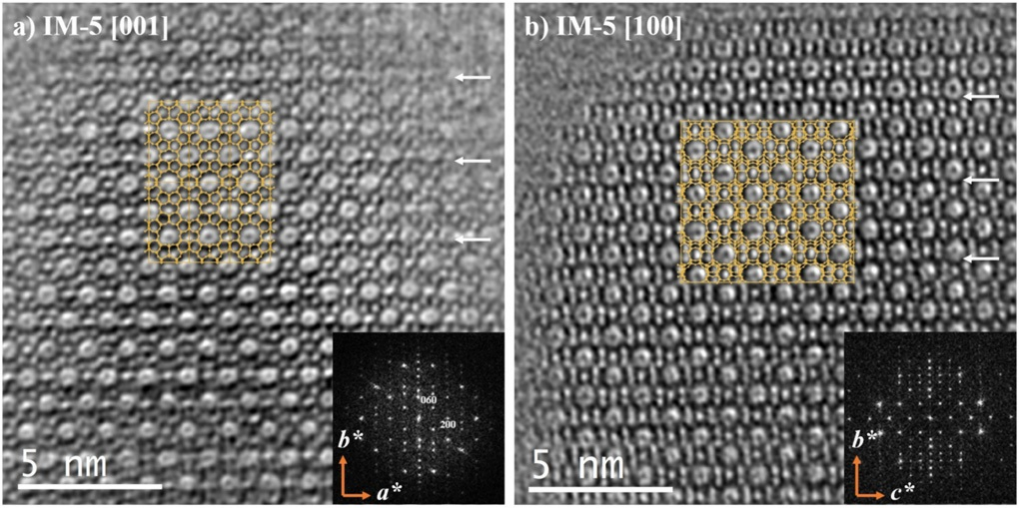
HR‐TEM images and the corresponding FD of calcined IM‐5 taken along [001] and [100]. Taken by JEM 3010 at 300 kV. The projected structure models were overlapped on the images.

Unambiguous determination of the local structural modulation was further proved from HR‐TEM images at the atomic level. The processed HRTEM images of **IMF** obtained by Ruan,^22^ by enhancing the signal to noise ratio (SNR) without imposing any symmetry constraints along [001] and [100] incidences are shown in Figure 10. In both images, contrast modulations are seen along the *b*‐axis with different periods (marked by arrows in Figure 10 a,b, respectively), which corresponds to the previous discussion on ED patterns (Figure 8 b). The observed modulations are equal to *b*/2, which means that there is no actual super lattice in the crystals but the intrinsic modulated repeated units in one unit cell of IMF. We believe this is the reason that Sun and co‐workers call them *pseudo* three‐fold super‐lattices. As for [001] incidence, the projected structure shows similarities to the **MFI** framework, which consists of pentasil chains (sheets for **IMF**) and the modulated contrasts appear every three pentasil chains, a half *b*.

The schematic models based on Baerlocher's structure solution is inserted showing good match with the observed images with both FDs as insets.

### 2.3 New advances

**Figure 11:**
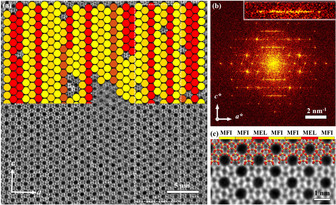
a) C_s_‐corrected STEM‐ADF observation. MFI/MEL crystal with **MEL** as red spheres. b) The corresponding FD is inserted at the top left, c) Closer observation from the dashed region of a) with the model overlaid. Reproduced with permission.^23^ Copyright 2020, Springer Nature.

With the implementation of spherical aberration (C_s_) correctors coupled with more and better electron detectors, lateral resolution is significantly improved for such beam sensitive materials. Very recently, Prashant et al.^23^ have succeeded in the synthesis of very well controlled **MFI** nanosheets with a lateral area of 200 nm × 200 nm and uniform thickness of only 3.2 nm along the *b*‐axis. The authors found that one to few unit cells intergrowths of **MEL** domains were inserted along the *a*‐axis in the **MFI** framework and extended along the *c*‐axis. Based on ultra‐high resolution C_s_‐corrected STEM data using an annular dark field detector (ADF), they identified the planar modulation distribution of these **MEL** domains, and showed that a fraction of nanosheets have a significant amount of **MEL** content (≈25 % by volume) while the majority of nanosheets are purely **MFI**. This work combined traditional SAED patterns collection and analysis with state‐of‐art EM observations, image treatment and data analysis (Figure 11). In here, an intergrowth of a **MEL** band was observed between the two **MFI** domains, which are in mirror relation to each other through the **MEL** band perpendicular to the *a*‐axis (Figure 11 a). They are marked with circles or belts in different colours. The corresponding FD is shown in Figure 11 b. A region corresponding to the diffuse streaks is enlarged and show inset corresponding to the 102 reflection (*h* + *l*=2*n* + 1), which indicates the presence of finite domains of **MFI** trapped between **MEL** layers along the *a*‐direction. A closer observation of the framework is shown in Figure 11 c, with the two frameworks denoted and the model superimposed.

The development of C_s_‐corrected STEM with various detectors, such as annular bright‐field (ABF), ADF and high‐angle annular dark‐field (HAADF) detectors, gives enhanced contrast from light elements and medium atomic number Z‐atoms to heavy Z‐atoms, respectively. Furthermore, cutting‐edge characterization of hetero‐atom or vacancies in the framework of zeolites with atomic resolution electron microscopes combined with advanced analytical methods plays more essential roles in the structural characterization of zeolites, especially for modulated structures that are largely related to the catalytic activities.

With the intention of utilizing the power and potential of advanced EM in the observation and analysis of nanoporous materials, we present data obtained from **MFI** crystals as a first step toward the characterization of point modulation. In this case, very thin areas were investigated, and the Si atoms of the framework were clearly differentiated by both ADF and ABF imaging.

**Figure 12:**
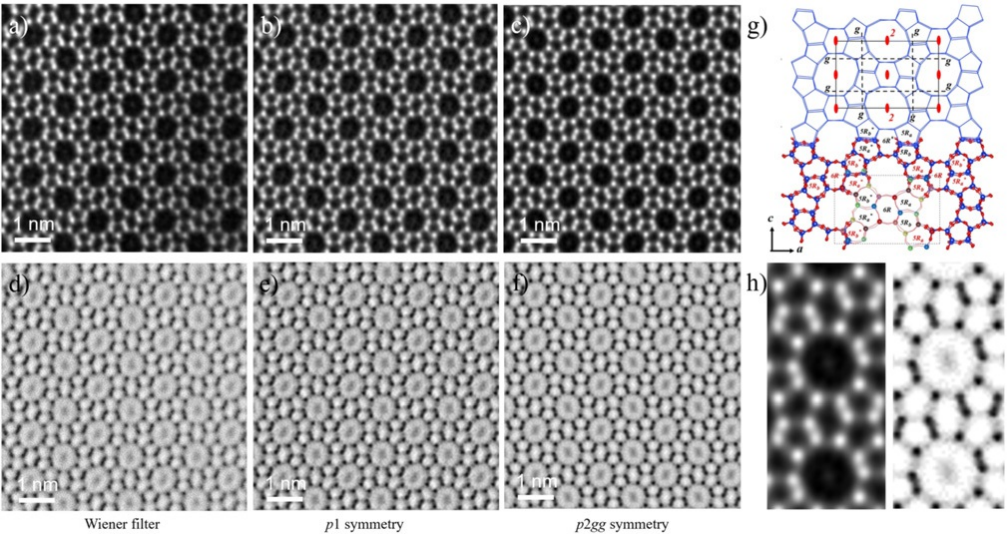
C_s_‐corrected STEM images of **MFI** on the [010]. a) ADF Wiener filtered image. b) ADF *p*1 symmetry averaged image. c) ADF *p*2*gg* symmetry averaged image. d) ABF Wiener filtered image. e) ABF *p*1 symmetry averaged image. f) ABF *p*2*gg* symmetry averaged image. g) Structural model, unit‐cell with the symmetry operations and the membered rings marked. h) Closer observation of the ADF and ABF *p*2*gg* data.

Figure 12 shows C_s_‐corrected **MFI** images along the *b*‐axis using both detectors. ADF and ABF can be differentiated as they produce reversed contrast images, with ABF similar to conventional TEM imaging. The ADF has been more widely used as this detector is more readily available, image acquisition is easier and the signal is sensitive to atomic number of the elements,^24^ which makes it suitable for analysing metals in light supports as zeolites.^25^ On the other hand, ABF is more sensitive to aberrations in the microscope and thus data acquisition is more challenging; however, it provides complementary information on light compounds, such as light cations and oxygen bridges. Figure 12 a–c shows the atomic‐resolution ADF data of the **MFI** framework in the [010] orientation (atoms appear in white), while the reversed contrast is obtained from the ABF detector (Figure 12 d–f). Because of the low SNR, images were Wiener filtered (Figures 12 a and 12 d for ADF and ABF respectively) allowing direct visualization of the four types of rings: 5Ra, 5Rb, 6R and 10R. To further extend the information limit, images were symmetry averaged by *p*1, just translational averaging by unit cell vectors *a* and *c* (Figure 12 b,e) and *p*2*gg* (Figure 12 f), which are the projected symmetry along the [010] orientation for *Pnma*. We can see particularly good match with the projected framework of **MFI** (Figure 12 g). Despite the excellent data reported in recent years by different groups, we consider that spatial resolution should be further increased to be able to discuss point defects as point modulations at least at the atomic level shown in Figure 12. Figure 12 h shows an enlargement of the *p*2*gg* micrographs, which clearly reveal the pentasil chains described earlier, and in the case of the ABF image, the visualization of oxygen bridges between Si atoms.

**Figure 13:**
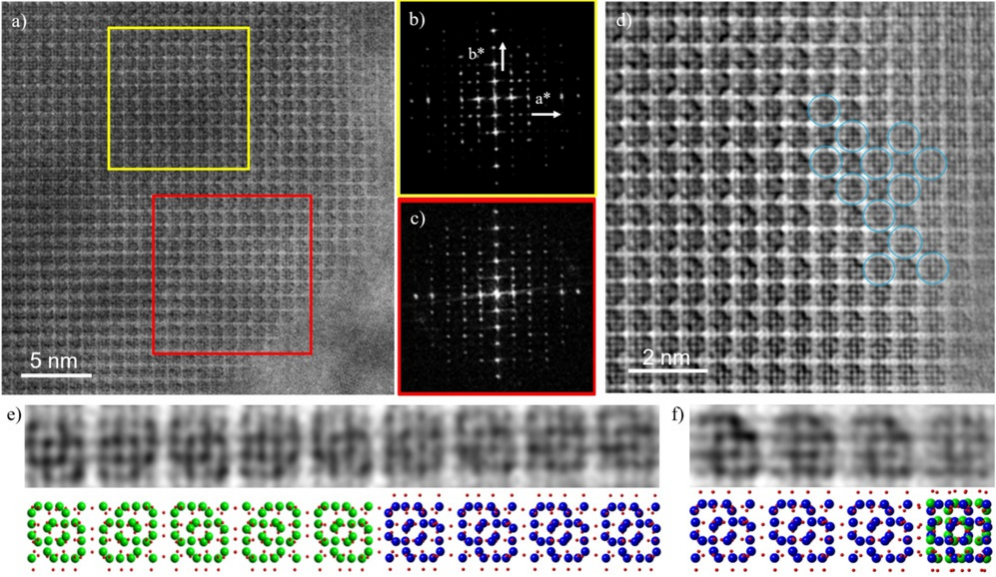
a) C_s_‐corrected STEM‐ABF along the [001] where the two areas of analysis are marked by yellow and red rectangles, together with their corresponding FDs (b and c). d) Closer observation with the modulations marked by blue circles. e) Magnified region of the nearly perfect modulation with the model shown below, green region corresponds to **MEL** while the blue one is **MFI**. f) Similar observation, where the two structures superimposed, its corresponding model appears below with the same color code. Oxygen atoms are always red spheres.

Earlier in this review, we mentioned that the best orientation to study the MFI/MEL modulation is [001], as it is the orientation in which the 5Rs are connected differs depending on the framework (Figure 4). In this sense, only Oshuna's work17c has shown this modulation experimentally for zeolite **MEL**. At that time, the spatial resolution was limited by aberrations in the lenses and if these modulations occur locally in a short range, they could be unnoticed. Based on atomic resolution observation along this projection (Figure 13), the structures can now be studied from membered rings to membered rings. Figure 13 a corresponds to the C_s_‐corrected STEM ABF micrograph of **MFI** along [001]; the two areas marked by yellow and red rectangles were further studied. From the FDs (Figure 13 b,c) we infer some variations respect to the ideal FD (Figure 4). FDs obtained from both regions show significant differences: while the red rectangle can be indexed as **MFI**, the yellow region does not strictly match either **MFI** or **MEL**. Magnified data are presented in Figure 13 d. The blue circles in Figure 13 d mark the local modulations in this crystal, which are formed as a result of co‐existence along the *c*‐axis of units from both **MFI** and **MEL**; therefore, the direction of the interconnected 5Rs cannot be distinguished as both orientations are overlaid along the same columns. From Figure 13 e and 13 f, this can be clearly appreciated. In Figure 13 e, a perfect, or nearly perfect transition between **MEL** and **MFI** is visualized, for a better understanding the model is presented below, where **MEL** units appear in green. On the other hand, in Figure 13 f, four units of the interconnected 5Rs are depicted, three of which correspond to pure **MFI** (blue), while the other is associated with the coexistence of both **MFI** and **MEL** along the same column (blue and green in the model).

These results, which were obtained from a typical **MFI** zeolite, raise the possibility that modulations between **MFI** and **MEL** may be more common than it has been reported. However, the fact that they occur at a local level suggests the possibility that they have not been noticed in some cases.

## 3 Conclusions

In this Minireview, we have summarized the relevant modulated structures in a few types of zeolites, from the simplest end‐members of the pentasil family MFI/MEL to SSZ‐57 with interesting pseudo super lattice and the most puzzling structure IM‐5. These examples illustrate a variety of local structural modulations including planar modulation, surface termination and intrinsic modulation in the framework, together with recent observations that reveal planar intergrowths of **MFI** and **MEL** at the atomic level.

With the most advanced C_s_ corrected EM techniques in combination with other characterization techniques, such as X‐ray diffraction and mathematical algorithms, it is nowadays possible to trace more precisely the local modulation in the framework, not only involving heavy atoms, but also light elements or even single heteroatoms. The direct observation of all framework atoms of Na‐LTA and atomic level substitution of Si by Fe in Fe‐MFI has already been reported.^26^

To locally characterize modulated structures of zeolites at the atomic level, the key challenges from the experimental point of view are: (i) how to overcome electron beam sensitivity of zeolite crystals in order to acquire more precise data, (ii) how to obtain highly crystalline and thin specimens with clean surfaces, (iii) how to obtain precise/reproducible 3d‐structural information through diffraction and imaging and (iv) how to develop a methodology to obtain 3d‐structure solution including local structure modulations such as point modulations.

Finally, in the near future, special efforts are expected be achieved to distinguish Si and Al directly and also to observe vacancy/point defects in a framework called “hydroxy‐nest” at the atomic level, which will facilitate the study of modulated point defects in the framework of structures that are related to dealumination processes, synthesis of mesoporous materials and so on.

## Conflict of interest

The authors declare no conflict of interest.

## Biographical Information

*Qing Zhang received her PhD degree in Laser Optics in 2011 from Institute of Physics in Chinese Academy of Sciences. After a postdoctoral fellowship at the Deutsches Elektronen‐Synchrotron (DESY) in Germany, she joined ShanghaiTech University as a research assistant in 2018. She has recently been a research associate working on characterization of various types of porous materials including both imaging and spectroscopy by using aberration‐corrected scanning/transmission electron microscopy*.

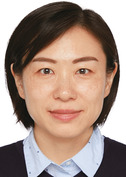

## Biographical Information

*Alvaro Mayoral studied chemistry at the University of Alcala (Spain) and obtained his PhD at the University of Birmingham. Since 2018, he is Research Associate Professor at ShanghaiTech University and from 2020; he is a Ramon y Cajal researcher at INMA‐CSIC. He is working on the development and application of new electron microscopy methods for beam sensitive nanoporous solids. He is also interested on metallic nanoparticles and other materials from the nanotechnology perspective, covering from fundamental aspects up to potential and industrial applications*.

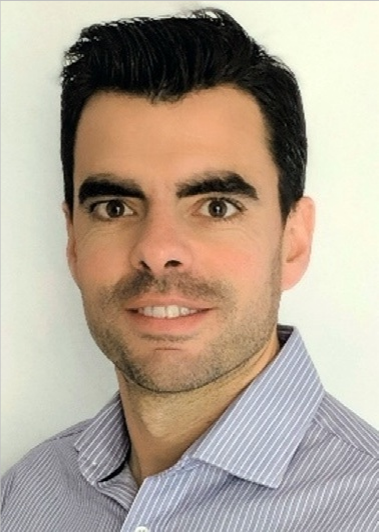

## Biographical Information

*Osamu Terasaki worked at Department of Physics, Tohoku Univ, Japan as a faculty member (1967–2003), and stayed at Cambridge Univ (1982–1984) and Lund Univ (1988.3–9) to start his research on fine structure of zeolites. He was one of the first Research Directors of National CREST project, Japan (1995–2000). OT was Prof of Structural Chemistry, Stockholm Univ (2003–2010), and KAIST, Korea (2009–2016). He is currently Director, CħEM, ShanghaiTech Univ. OT received Friendship Award from China, the Donald Breck Award and Humboldt‐Research Award*.

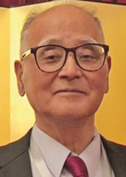

## References

[1] W. Löwenstein , Am. Mineral. 1954, 39, 92–96.

[2] R. E. Fletcher , S. Ling , B. Slater , Chem. Sci. 2017, 8, 7483–7491. 29163901 10.1039/c7sc02531a PMC5676096

[3] Structure Commission of the International Zeolite Association (IZA-SC). Available from: http://www.iza-structure.org/.

[4a] G. T. Kokotailo , S. L. Lawton , D. H. Olson , W. M. Meier , Nature 1978, 272, 437–438;

[4b] G. T. Kokotailo , P. Chu , S. L. Lawton , W. M. Meier , Nature 1978, 275, 119–120.

[5a] A. P. Giddy , M. T. Dove , G. S. Pawley , V. Heine , Acta Crystallogr. Sect. A 1993, 49, 697–703;

[5b] K. D. Hammonds , M. T. Dove , A. P. Giddy , V. Heine , Am. Mineral. 1994, 79, 1207–1209.

[6] G. Perego , M. Cesari , G. Allegra , J. Appl. Crystallogr. 1984, 17, 403–410.

[7] G. Millward , J. Thomas , O. Terasaki , D. Watanabe , Zeolites 1986, 6, 91–95.

[8] A. Turrina , R. Garcia , A. E. Watts , H. F. Greer , J. Bradley , W. Zhou , P. A. Cox , M. D. Shannon , A. Mayoral , J. L. Casci , Chem. Mater. 2017, 29, 2180–2190.

[9] J. M. Thomas , G. R. Millward , J. Chem. Soc. Chem. Commun. 1982, 1380–1383.

[10a] Y. Li , X. Li , J. Liu , F. Duan , J. Yu , Nat. Commun. 2015, 6, 8328; 26395233 10.1038/ncomms9328 PMC4667440

[10b] J. Čejka , R. E. Morris , P. Nachtigall , Zeolites in Catalysis: Properties and Applications, CPI Group (UK), Croydon, 2017.

[11a] V. Alfredsson , Micron Microsc. Acta 1992, 23, 133–134;

[11b] O. Terasaki , T. Ohsuna , Catal. Today 1995, 23, 201–218.

[12] V. Alfredsson , T. Ohsuna , O. Terasaki , J.-O. Bovin , Angew. Chem. Int. Ed. Engl. 1993, 32, 1210–1213;

[13] T. Ohsuna , O. Terasaki , D. Watanabe , M. W. Anderson , S. W. Carr , Chem. Mater. 1994, 6, 2201–2204.

[14] O. Terasaki , K. Yamazaki , J. M. Thomas , T. Ohsuna , D. Watanabe , J. V. Sanders , J. C. Barry , Nature 1987, 330, 58–60.

[15] T. Ohsuna , B. Slater , F. Gao , J. Yu , Y. Sakamoto , G. Zhu , O. Terasaki , D. E. W. Vaughan , S. Qiu , C. R. A. Catlow , Chem. Eur. J. 2004, 10, 5031–5040. 15372583 10.1002/chem.200306064

[16] G. R. Millward , S. Ramdas , J. M. Thomas , M. T. Barlow , J. Chem. Soc. Faraday Trans. 2 1983, 79, 1075–1082.

[17a] O. Terasaki , J. M. Thomas , G. R. Millward , D. Watanabe , Chem. Mater. 1989, 1, 158–162;

[17b] O. Terasaki , T. Ohsuna , H. Sakuma , D. Watanabe , Y. Nakagawa , R. C. Medrud , Chem. Mater. 1996, 8, 463–468;

[17c] T. Ohsuna , O. Terasaki , Y. Nakagawa , S. I. Zones , K. Hiraga , J. Phys. Chem. B 1997, 101, 9881–9885.

[18] Z. Liu , N. Fujita , O. Terasaki , T. Ohsuna , K. Hiraga , M. A. Camblor , M. J. Díaz-Cabañas , A. K. Cheetham , Chem. Eur. J. 2002, 8, 4549–4556. 12355545 10.1002/1521-3765(20021004)8:19<4549::AID-CHEM4549>3.0.CO;2-Z

[19] C. Baerlocher , T. Weber , L. B. McCusker , L. Palatinus , S. I. Zones , Science 2011, 333, 1134. 21868674 10.1126/science.1207466

[20] S. I. Zones , J. Ruan , S. Elomari , O. Terasaki , C. Y. Chen , A. Corma , Solid State Sci. 2011, 13, 706–713.

[21a] C. Baerlocher , F. Gramm , L. Massuger , L. B. McCusker , Z. He , S. Hovmoller , X. Zou , Science 2007, 315, 1113–1116; 17322057 10.1126/science.1137920

[21b] J. Sun , Z. He , S. Hovmöller , X. Zou , F. Gramm , C. Baerlocher , L. B. McCusker , Z. Kristallogr. 2010, 225, 77–85.

[22] J. Ruan, PHD thesis, Stockholm University **2008**.

[23] P. Kumar , D. W. Kim , N. Rangnekar , H. Xu , E. O. Fetisov , S. Ghosh , H. Zhang , Q. Xiao , M. Shete , J. I. Siepmann , T. Dumitrica , B. McCool , M. Tsapatsis , K. A. Mkhoyan , Nat. Mater. 2020, 19, 443–449. 32094494 10.1038/s41563-019-0581-3

[24a] A. Mayoral , S. Mejía-Rosales , M. M. Mariscal , E. Pérez-Tijerina , M. José-Yacamán , Nanoscale 2010, 2, 2647–2651; 20944844 10.1039/c0nr00498g

[24b] A. Mayoral , D. Llamosa , Y. Huttel , Chem. Commun. 2015, 51, 8442–8445. 10.1039/c5cc00774g 25719945

[25a] A. Mayoral , T. Carey , P. A. Anderson , A. Lubk , I. Diaz , Angew. Chem. Int. Ed. 2011, 50, 11230–11233; 10.1002/anie.201105450 21956896

[25b] A. Mayoral , R. M. Hall , R. Jackowska , J. E. Readman , Angew. Chem. Int. Ed. 2016, 55, 16127–16131; 10.1002/anie.201609094 27882639

[25c] A. Mayoral , J. E. Readman , P. A. Anderson , J. Phys. Chem. C 2013, 117, 24485–24489;

[25d] C. Li , Q. Zhang , A. Mayoral , ChemCatChem 2020, 12, 1248–1269.

[26] A. Mayoral , Q. Zhang , Y. Zhou , P. Chen , Y. Ma , T. Monji , P. Losch , W. Schmidt , F. Schüth , H. Hirao , J. Yu , O. Terasaki , Angew. Chem. Int. Ed. 2020, https://doi.org/10.1002/anie.202006122; *Angew. Chem* **2020**, https://doi.org/10.1002/ange.202006122.

